# mTOR inhibitors sensitize thyroid cancer cells to cytotoxic effect of vemurafenib

**DOI:** 10.18632/oncotarget.4052

**Published:** 2015-05-09

**Authors:** Elyse K. Hanly, Robert B. Bednarczyk, Neha Y. Tuli, Augustine L. Moscatello, H. Dorota Halicka, Jiangwei Li, Jan Geliebter, Zbigniew Darzynkiewicz, Raj K. Tiwari

**Affiliations:** ^1^ Department of Microbiology and Immunology, New York Medical College, Valhalla, NY, USA; ^2^ Department of Otolaryngology/Head and Neck Surgery, New York Medical College, Valhalla, NY, USA; ^3^ Department of Pathology, New York Medical College, Valhalla, NY, USA

**Keywords:** vemurafenib, mTOR, thyroid cancer, cytotoxic effect, drug resistance

## Abstract

Treatment options for advanced metastatic thyroid cancer patients are limited. Vemurafenib, a BRAFV600E inhibitor, has shown promise in clinical trials although cellular resistance occurs. Combination therapy that includes BRAFV600E inhibition and avoids resistance is a clinical need. We used an *in vitro* model to examine combination treatment with vemurafenib and mammalian target of rapamycin (mTOR) inhibitors, metformin and rapamycin. Cellular viability and apoptosis were analyzed in thyroid cell lines by trypan blue exclusion and TUNEL assays. Combination of vemurafenib and metformin decreased cell viability and increased apoptosis in both BCPAP papillary thyroid cancer cells and 8505c anaplastic thyroid cancer cells. This combination was also found to be active in vemurafenib-resistant BCPAP cells. Changes in expression of signaling molecules such as decreased mTOR expression in BCPAP and enhanced inhibition of phospho-MAPK in resistant BCPAP and 8505c were observed. The second combination of vemurafenib and rapamycin amplified cell death in BCPAP cells. We conclude that combination of BRAFV600E and mTOR inhibition forms the basis of a treatment regimen that should be further investigated in *in vivo* model systems. Metformin or rapamycin adjuvant treatment may provide clinical benefits with minimal side effects to *BRAFV600E*-positive advanced thyroid cancer patients treated with vemurafenib.

## INTRODUCTION

Most cases of thyroid cancer are treated successfully with surgical resection, radioiodine, and thyroid hormone therapy. However, there are few treatment options available for patients with advanced disease including radioiodine-resistant and metastatic differentiated thyroid cancer and anaplastic thyroid cancer. Only 30% of patients with radioiodine-refractory differentiated thyroid cancer and pulmonary metastasis survive 5 years after diagnosis [1]. In addition, anaplastic thyroid cancer patients face an average life expectancy of less than one year. Recent progress in the treatment of advanced thyroid cancer patients includes targeted therapy. In 2013, the kinase inhibitor sorafenib became the first drug since doxorubicin to be FDA-approved in the past 40 years for treatment of radioiodine-resistant metastatic well-differentiated thyroid cancer [1]. More recently, a second kinase inhibitor lenvatinib was approved. Sorafenib and lenvatinib both inhibit several protein kinases including vascular endothelial growth factor receptor (VEGFR) and platelet-derived growth factor receptor (PDGFR). Sorafenib also inhibits RAF kinases and lenvatinib inhibits fibroblast growth factor receptor (FGFR), RET, and KIT. Both drugs improved progression-free survival compared to placebo in phase 3 trials [2, 3]. Other targeted agents currently in clinical trials for thyroid cancer include BRAFV600E inhibitors such as vemurafenib.

The *BRAFV600E* mutation is found in approximately one-half of papillary thyroid cancers and one-fourth of anaplastic thyroid cancers and is associated with poor prognosis [4-6]. The rationale behind targeting BRAFV600E kinase is that this protein is specific to cancer cells and drives the growth-promoting MAPK pathway. Thyroid cancer cells become dependent on BRAFV600E constitutive activation for growth, survival, and tumor progression. Vemurafenib binds selectively to the active site of BRAFV600E protein, which differs in conformation from wild-type BRAF, and inhibits downstream MAPK signaling [7]. Short-term treatment with BRAFV600E inhibitors has drastic anti-proliferative effects on mutated thyroid cancer cells including induction of apoptosis [8-10]. The drug vemurafenib has also recently proved to be useful in treating advanced thyroid cancer patients in clinical trials.

In an earlier study, one patient with metastatic thyroid cancer experienced a partial response including reduced pulmonary lesions after treatment with vemurafenib, and the two other patients had stable disease [11]. Additionally, two individual case reports documented tumor regression in response to vemurafenib in a patient with anaplastic thyroid cancer and a patient with advanced papillary thyroid cancer [12, 13]. Brose *et al*. presented results of an ongoing phase II clinical study for vemurafenib in patients with metastatic or unresectable *BRAFV600E*-positive papillary thyroid cancer at the 2013 ECCO/ESMO/ESTRO Annual Meeting. These results also demonstrate an anti-tumor effect thus far, especially in those patients who have not been treated with other tyrosine kinase inhibitors. Off-label use of vemurafenib for 17 patients with *BRAFV600E*-positive advanced papillary thyroid cancer was reviewed retrospectively and found to be effective and well-tolerated [14].

While these studies collectively point out the therapeutic potential for vemurafenib, as a single targeted therapy it will likely not be able to prevent drug resistance and disease progression in the long term. Combination therapy including BRAFV600E inhibitors is a promising option to delay or prevent resistance and further improve therapy for *BRAFV600E*-positive thyroid cancer patients. Researchers are now investigating combinations of targeted inhibitors, including dabrafenib (BRAFV600E inhibitor) and lapatinib (HER2/neu and EGFR inhibitor) in refractory and inoperable *BRAFV600E*-positive thyroid cancer. HER2/HER3 dimers were found to be increased in thyroid cells after vemurafenib treatment and are capable of activating several intracellular signaling pathways [15]. It is likely that activation of multiple growth-promoting signaling pathways including MAPK and PI3K/Akt contributes to development of vemurafenib resistance. One study concluded that BRAF inhibition resistance pathways converge to regulate translation of mRNAs through formation of the eIF4F initiation complex [16]. Since mammalian target of rapamycin (mTOR) is a major regulator of translation via eIF4F initiation complex formation and receives input from cell signaling pathways, it is also a rational target for combination therapy.

In this study we investigated the combination of vemurafenib and mTOR inhibitors, metformin and rapamycin, which has not yet been explored in thyroid cancer. Evidence for anti-tumor effects of metformin alone has been accruing for several types of cancer, although exact mechanisms are yet to be determined and most studies involve diabetic patients. One study found that treatment with metformin in diabetic patients with thyroid cancer resulted in smaller tumor size and greater chance of complete remission [17]. Studies in the laboratory have also confirmed growth-inhibitory effects of metformin on thyroid cancer cells [18, 19]. Metformin caused decreased cell viability and increased apoptosis in BCPAP and BHP10-3SC papillary thyroid cells and inhibited BHP10-3SC tumor growth in nude mice. These effects were attributed to activation of AMP kinase and inhibition of growth-promoting Akt and mTOR [19]. Along with inhibition of mTOR signaling, there are several mechanisms proposed for anti-cancer effects of metformin including STAT3 inhibition leading to induction of apoptosis and effects on cancer-related miRNAs [20]. Both vemurafenib and metformin appear to have anti-cancer effects in thyroid cancer, and it is possible that adjuvant metformin therapy would not significantly increase toxicity in vemurafenib-treated patients due to its high safety profile. Thus, we investigated effects of combination metformin and vemurafenib in thyroid cancer cells, hypothesizing enhanced cytotoxic effects.

We also investigated the potential combination of vemurafenib and rapamycin in thyroid cancer cells. Rapamycin is an immunosuppressant used to prevent organ transplant rejection, especially in kidney transplants. It has also been researched as an anti-cancer agent due to its anti-proliferative effects which are more potent than its immunosuppressive effects in terms of tumor growth [21]. Rapamycin forms a complex with cytosolic FK-binding protein 12 (FKBP12) and this complex serves as a direct and specific inhibitor of mTOR [22-26]. The rapamycin:FKBP12 complex inhibits the signal transduction ability of mTOR, preventing activation of multiple targets required for progression through cell cycle and resulting in G1 phase arrest. One phase II trial evaluated the efficacy and safety of everolimus, a rapamycin derivative, in advanced thyroid cancers and determined that the drug had some clinical benefit although further study is needed [27]. Inhibition of mTOR signaling with rapamycin in combination with inhibition of the driver mutated kinase BRAFV600E with vemurafenib in thyroid cancer may result in more profound anti-proliferative effects. We hypothesized that the combination treatment of vemurafenib with rapamycin, similar as with metformin, may also lead to enhanced cytotoxic effects in thyroid cancer cell lines.

## RESULTS

### Metformin alone and the metformin-vemurafenib combination treatment significantly decrease cell viability in *BRAFV600E*-positive papillary and anaplastic thyroid cancer cells

**Figure 1:**
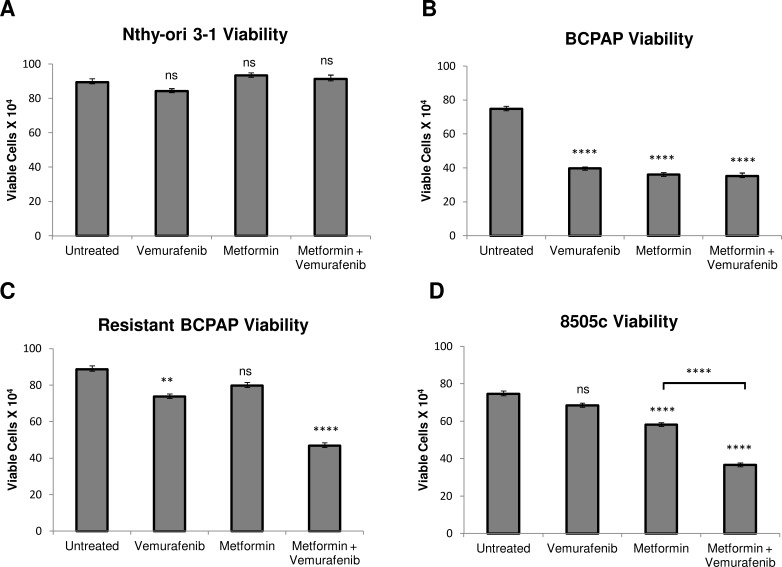
Effects of metformin and vemurafenib on cell viability Nthy-ori 3-1 **A.**, BCPAP **B.**, resistant BCPAP **C.**, and 8505c **D.** were subjected to a trypan blue exclusion assay after treating cells with +/− 2 mM metformin for 24 h prior to +/− 10 μM vemurafenib treatment for 36 h. Live cells were counted as the cells unable to take up trypan blue dye. Data from at least two independent experiments performed in triplicate are expressed as mean +/− SEM. *P < 0.05, **P < 0.01, ***P < 0.001, ****P < 0.0001.

We measured viability of several thyroid cell lines after treatment with vemurafenib, metformin, and the combination. As expected*, BRAFV600E*-positive BCPAP papillary thyroid cancer cells were susceptible to vemurafenib in terms of viability, which was decreased to 50% that of untreated (Figure 1B). In addition, treatment with metformin alone and with combination treatment of vemurafenib and metformin decreased frequency of viable BCPAP cells to approximately 50% of untreated cells (Figure 1B). Although 8505c anaplastic thyroid cancer cells were unaffected by treatment with vemurafenib, metformin alone decreased their viability significantly to 75% that of untreated. The metformin-vemurafenib combination further reduced 8505c frequency of live cells to 50% of untreated cells, and the number of viable cells was significantly lower than viable cells counted after treatment with metformin alone (Figure 1D). Nthy-ori 3-1 *braf*-wild-type thyroid cells demonstrated no significant changes in cell viability in response to any of the treatments compared to untreated (Figure 1A).

### Metformin-vemurafenib combination treatment significantly decreases viability in vemurafenib-resistant BCPAP cells

Another cell line evaluated was resistant BCPAP, which was created in the laboratory by exposing BCPAP cells to increasing concentrations of vemurafenib. As expected, these cells were relatively resistant to treatment with vemurafenib alone compared to normal BCPAP (Figure 1B, 1C). Interestingly, vemurafenib-resistant BCPAP cells also appeared to be completely resistant to metformin, as there was no significant change in their viability in response to treatment with this drug as a single agent. However, the cell viability decreased significantly, to about 50% of untreated cells after treatment with the combination of metformin and vemurafenib (Figure 1C).

### Metformin-vemurafenib combination treatment increases frequency of apoptosis in BCPAP and 8505c cells

**Figure 2:**
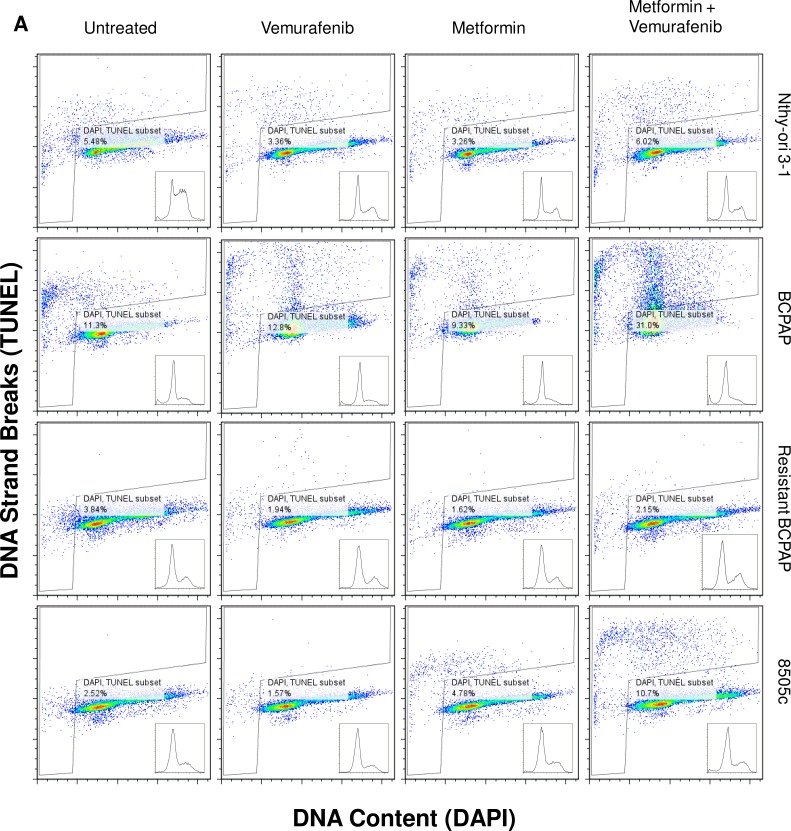

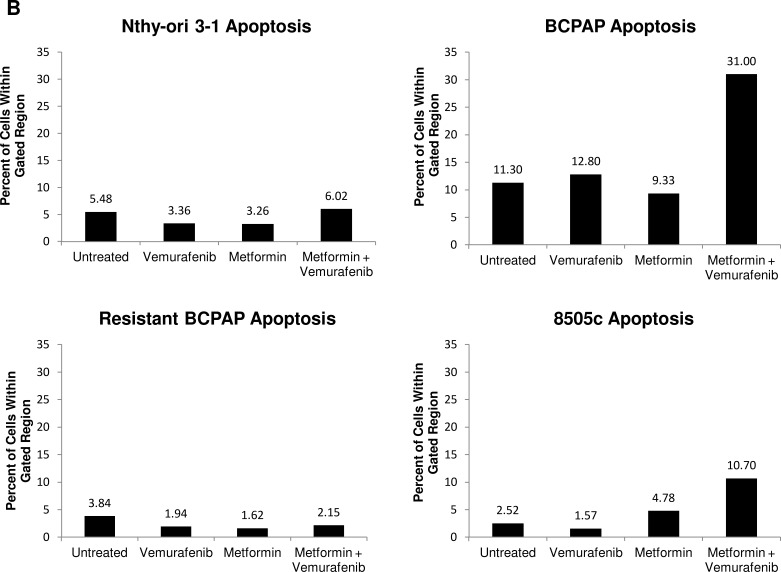
Apoptosis detected after metformin and vemurafenib treatment in BCPAP and 8505c cells Cell lines were treated with +/− 2 mM metformin for 24 h prior to +/− 10 μM vemurafenib for 36 h. **A.** Collected cells were subjected to APO-BrdU TUNEL staining and analyzed by flow cytometry. DNA content detected by DAPI staining is represented on the x-axis. The y-axis is a measure of BrdU incorporated into DNA strand breaks and immunocytochemically detected by Alexa Fluor 488 dye. Gated regions indicate apoptotic cells. **B.** Percentages of apoptotic cells determined in A are expressed in bar graphs for comparison.

Apoptosis, or programmed cell death, was measured in each of the thyroid cell lines following treatment with vemurafenib, metformin, and the combination. Terminal Deoxynucleotide Transferase dUTP Nick End Labeling (TUNEL) was used to identify DNA strand breaks that are characteristic of apoptotic cells [28]. In Figure 2, cells within the gated areas represent apoptotic cells with fragmented DNA (Figure 2A) and this percentage is also represented graphically (Figure 2B). In the BCPAP papillary thyroid cancer cells, some degree of apoptosis occurred in the untreated group (11.3%) revealing the background level of cell death under experimental conditions. Between the vemurafenib-treated sample and the metformin-treated sample the number of apoptotic cells detected by this assay varied only slightly, at 12.8% and 9.33% of total cells, respectively. However, in the combination treatment group, BCPAP cells demonstrated increased frequency of apoptosis with 31.0% of cells within the gated area (Figure 2A, 2B).

Percentage of apoptotic cells changed very little between untreated (2.5%) and vemurafenib-treated (1.6%) samples of 8505c anaplastic thyroid cells. Metformin-treated 8505c cells demonstrated a slight increase in apoptosis (4.8%). As in the BCPAP cells, a greater than additive effect in terms of induction of apoptosis was observed in the 8505c cells in response to combination treatment with metformin and vemurafenib. Drug combination treatment resulted in 10.7% apoptotic cells (Figure 2A, 2B). Resistant BCPAP cells in this assay showed little variability in apoptosis, indicating that although combination treatment with metformin and vemurafenib inhibits growth of these cells (Figure 1C), it does not cause overt apoptosis that can be detected by the TUNEL assay [28] (Figure 2A, 2B).

### Metformin and combination treatment modulate signaling molecules MAPK and mTOR

**Figure 3:**
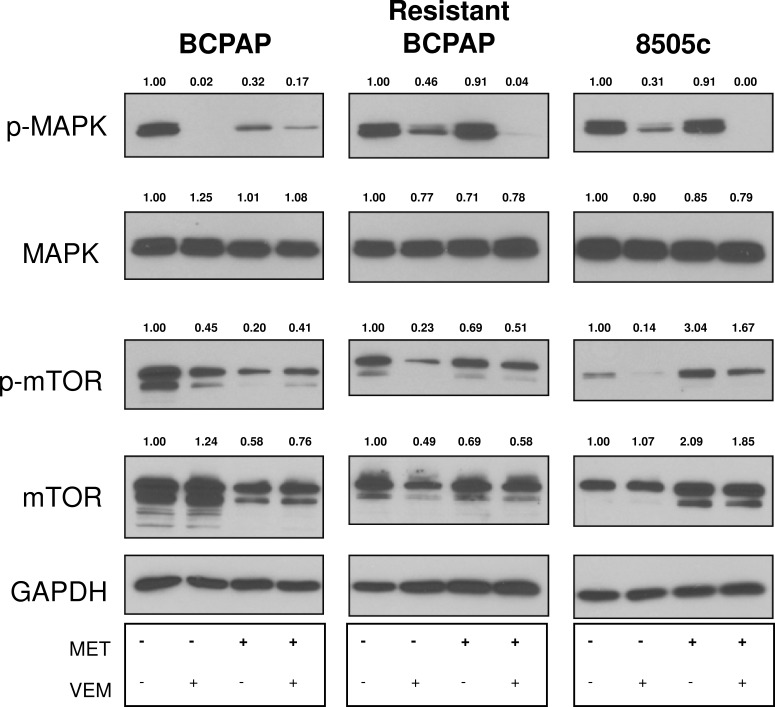
Effects of metformin and vemurafenib on signaling molecules Whole cell protein was collected from thyroid cancer cells that were treated with +/− 2 mM metformin for 24 h and +/− 10 μM vemurafenib for an additional 24 h. Protein samples (10 μg per lane) were subjected to SDS-PAGE followed by western blot analysis for p-MAPK (42/44 kD), MAPK (42/44 kD), p-mTOR (289 kD) and mTOR (289 kD). GAPDH (37 kD) was used as a loading control. Numbers represent densitometry values after normalizing to GAPDH and the untreated samples.

Effects of metformin on thyroid cancer cell signaling pathways are largely unknown. We analyzed expression of MAPK and mTOR in response to metformin and combination treatment to determine whether signaling effects could play a role in mediating anti-proliferative effects. Co-treatment with metformin and vemurafenib led to enhanced inhibition of phospho-MAPK expression in resistant BCPAP and 8505c cells compared to vemurafenib alone (Figure 3). This effect was not observed in normal BCPAP. Effects on mTOR also varied among thyroid cancer cell lines, as total mTOR expression decreased in response to metformin in BCPAP and increased in 8505c anaplastic cells (Figure 3).

### Rapamycin-vemurafenib combination treatment significantly decreases viability compared to vemurafenib alone in BCPAP cells

**Figure 4:**
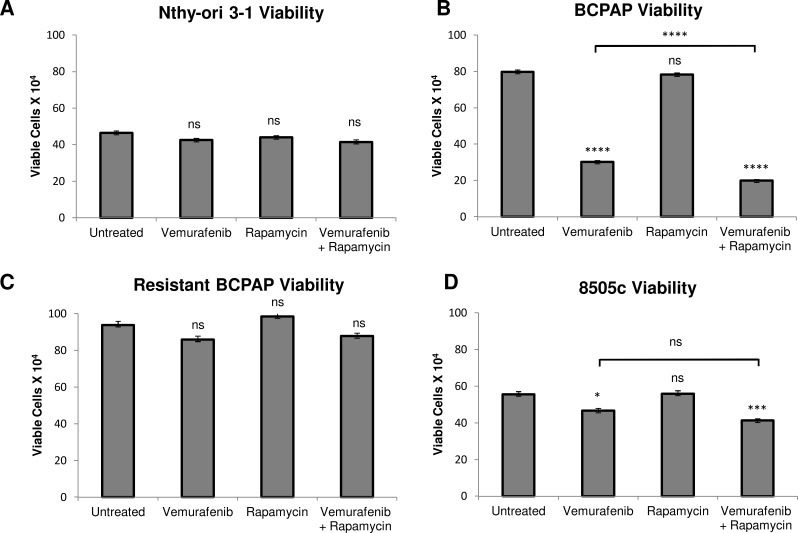
Effects of rapamycin and vemurafenib on cell viability Nthy-ori 3-1 **A.**, BCPAP **B.**, resistant BCPAP **C.**, and 8505c **D.** were subjected to a trypan blue exclusion assay after treatment with +/− 1 μM rapamycin and +/− 10 μM vemurafenib for 36 h. Live cells were counted as cells unable to take up trypan blue dye. Data from at least two independent experiments performed in triplicate are expressed as mean +/− SEM. *P < 0.05, **P < 0.01, ***P < 0.001, ****P < 0.0001.

We investigated a second possible combination therapy of rapamycin and vemurafenib in thyroid cells. Rapamycin serves as a direct specific inhibitor of mTOR. In a series of viability assays, rapamycin treatment alone had no significant effect on cell viability in all thyroid cell lines analyzed (Figure 4). Interestingly, the combination of rapamycin and vemurafenib decreased viability significantly in BCPAP papillary thyroid cancer cells, even compared to the vemurafenib-treated sample. Combination treatment resulted in a profound viability decrease to only 25% that of the untreated sample (Figure 4B). In 8505c, the difference in viability between vemurafenib-treated and combination-treated samples was not significant (Figure 4D). The resistant BCPAP and Nthy-ori 3-1 cells were not susceptible to rapamycin or the combination treatment in terms of viability (Figure 4A, 4C).

### Rapamycin-vemurafenib combination treatment increases frequency of apoptosis in BCPAP cells

**Figure 5:**
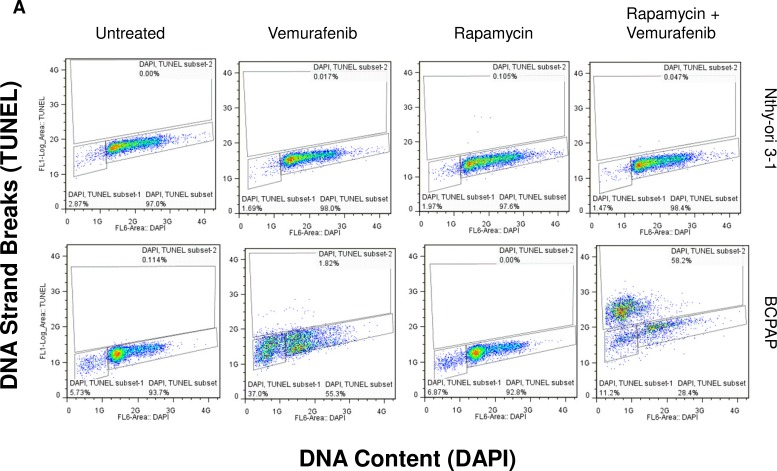

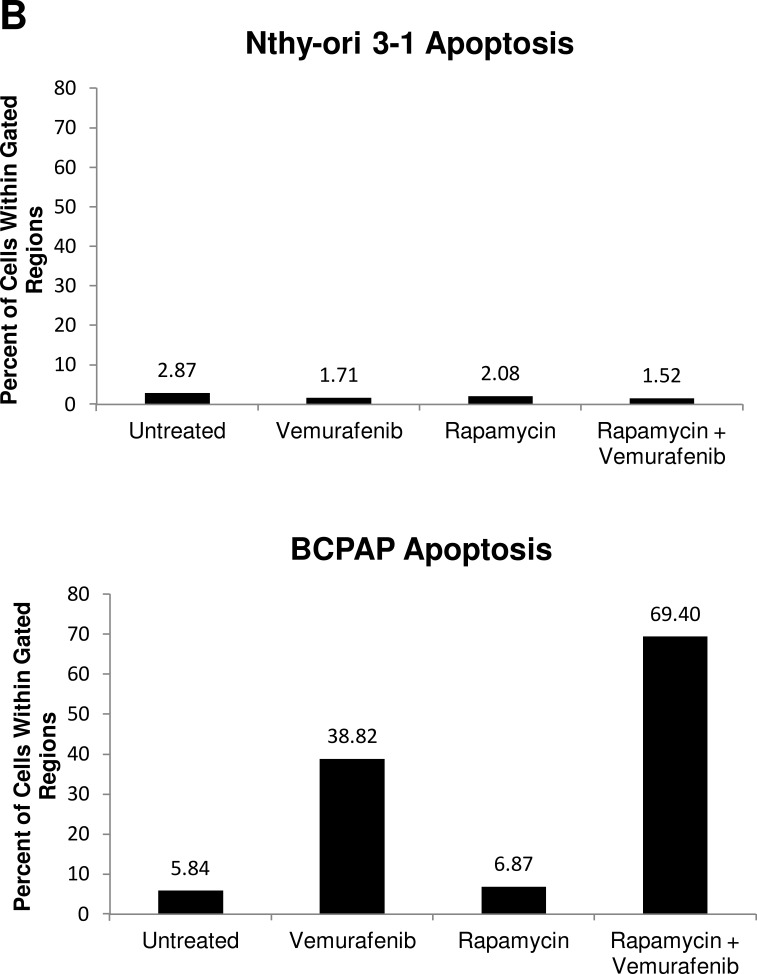

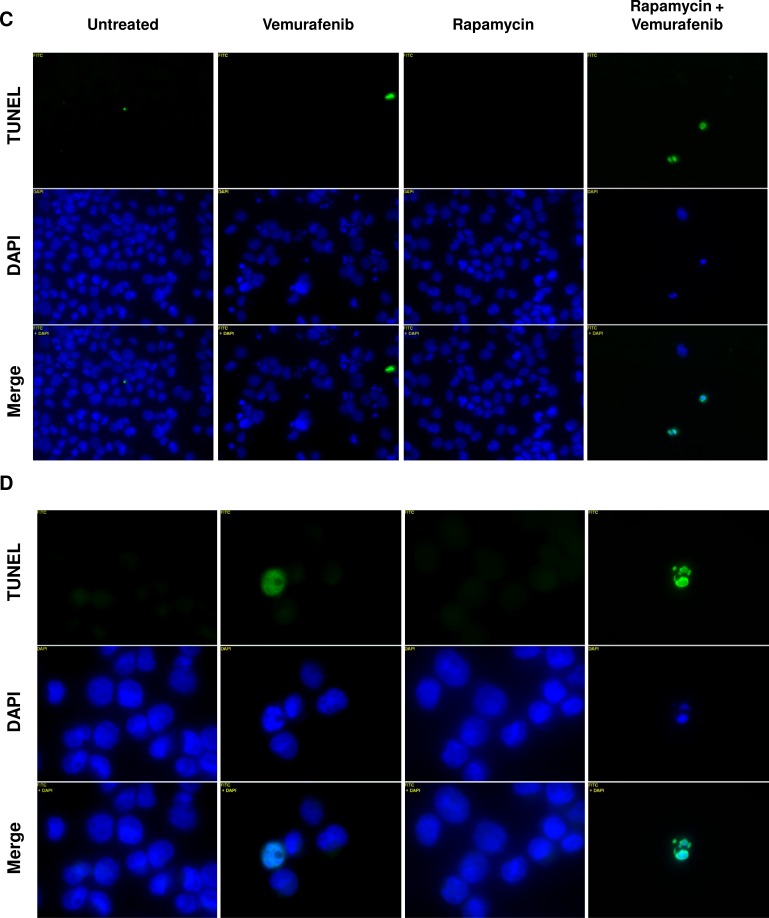
Apoptosis detected after rapamycin and vemurafenib treatment in BCPAP cells BCPAP and Nthy-ori 3-1 cells were treated with +/− 1 μM rapamycin and +/− 10 μM vemurafenib for 36 hours. **A.** Collected cells were subjected to APO-BrdU TUNEL staining and analyzed by flow cytometry. DNA content detected by DAPI is represented on the x-axis. The y-axis is a measure of BrdU incorporated into DNA strand breaks and immunocytochemically detected by Alexa Fluor 488 dye. Some apoptotic cells are also recognized based on their fractional DNA content (“sub-G1” cells, prominent in BCPAP, vemurafenib alone). Gated regions indicate apoptotic cells. **B.** Percentages of apoptotic cells determined in A are expressed in bar graphs for comparison. **C., D.** Samples were mounted onto microscope slides for viewing. Pictures were taken at 20x **C.** and 100x **D.** (Blue = DAPI staining for DNA content, Green = APO-BrdU TUNEL staining for DNA strand breaks).

To further investigate cytotoxic effects in BCPAP cells in response to rapamycin and vemurafenib, we conducted another TUNEL-based assay to evaluate apoptosis. We found the percentage of apoptotic cells increased in both vemurafenib and combination treatment groups compared to untreated cultures (Figure 5A, 5B). Concordant with results of viability assays, combination treatment caused a greatly enhanced frequency of apoptosis compared to vemurafenib treatment alone. Percentage of apoptotic cells increased from about 40% with vemurafenib alone to 70% with the combination of rapamycin and vemurafenib (Figure 5B). We also detected a large population of cells with high TUNEL staining in the combination group seen in Figure 5A, indicating a relative abundance of exposed 3′-hydroxyl ends of the DNA strand breaks compared to cells treated with vemurafenib alone. In the latter case apoptotic cells were identified as cells with fractional DNA content (“sub-G1” cells). Microscopically, vemurafenib-treated BCPAP cells show distinct signs of apoptosis including chromatin condensation and nuclear fragmentation. The drug combination-treated cells visualized support the finding of increased TUNEL-positive staining measured by flow cytometry (Figure 5C). In this assay, there were few apoptotic cells detected in the untreated and rapamycin-treated BCPAP cells (Figure 5A, 5B). *BRAF-* wild-type Nthy-ori 3-1 cells were used as a negative control and did not demonstrate appreciable apoptosis in any treatment group (Figure 5A, 5B).

## DISCUSSION

The use of targeted molecular therapy will likely continue to expand as cancer treatments become personalized and additional clinically relevant targets are discovered. Potent inhibitors of BRAFV600E, such as vemurafenib, show great promise for treatment of *BRAFV600E*-positive thyroid cancer. *BRAFV600E* mutation is also found in melanoma and colorectal cancer. Studies involving metastatic melanoma patients established that vemurafenib caused tumor regression and significantly reduced risk of death compared to standard dacarbazine treatment, although patients often developed resistance and disease progression resumed after 6-8 months [29, 30]. In cases of *BRAFV600E*-positive colorectal cancers, inhibition of BRAFV600E alone was ineffective, largely due to feedback activation of epidermal growth factor receptor (EGFR) [31-34]. Recent research in both melanoma and colorectal cancer revealed that combination therapy including BRAFV600E inhibition may provide greater benefits. Clinical studies thus far include targeting molecules that fall directly within the canonical MAPK pathway, such as MEK in melanoma [35, 36] and molecules that are membrane receptors activated due to feedback mechanisms, such as EGFR in colorectal cancer [37]. Immune checkpoint inhibitors combined with targeted therapy are also being considered in melanoma [38]. In this study, we investigated the combination of BRAFV600E inhibition and mTOR inhibition in thyroid cancer cells. The two mTOR inhibitors we chose are both clinically available with known pharmacokinetics and with somewhat differing mechanisms of action.

Metformin transiently inhibits the respiratory chain complex 1 in mitochondria and this leads to an increase in ratio of AMP to ATP, thereby triggering activation of AMP-activated protein kinase (AMPK): the latter subsequently leads to mTOR inhibition [39-42]. Actions of metformin in cancer cells may also include reducing expression of key enzymes involved in fatty acid synthesis as well as membrane tyrosine kinase receptors [42-44]. The combination of vemurafenib and metformin is an example of a strategy to combine therapies that target both the driver mutation *BRAFV600E* and more broadly inhibit critical cellular signaling pathways related to metabolism. It has been proposed that resistance to targeted therapy can be mediated by rewiring of cellular metabolism [45]. If changes in metabolism contribute to resistance, co-treatment with agents that alter cellular energy balance may hinder development of resistance or be effective against resistant tumors, as suggested by our results in thyroid cancer cells. Further elucidation of the link between driver mutations, resistance to targeted therapy and metabolism may build upon new avenues for cancer therapies.

Combination therapy with metformin and vemurafenib proved to be effective at inhibiting all thyroid cancer cell lines analyzed, including BCPAP, resistant BCPAP, and 8505c. Induction of apoptosis was greatest in BCPAP and also occurred in 8505c. We observed a general pattern of decreased viability corresponding to increased apoptosis. However, the combination treatment increased BCPAP apoptosis compared to vemurafenib alone without a significant change in viability between the two groups. A likely explanation is that decreases in cell viability may indicate decreased growth rate in response to treatment, in addition to cell death. In BCPAP, the decrease in viability after combination treatment can be attributed largely to the percentage of apoptotic cells detected by TUNEL. In the vemurafenib-treated group, decreased viability may indicate slower growth rate or the presence of disrupted cells without DNA strand breaks. In regard to resistant BCPAP cells, viability decreased significantly without a corresponding increase in apoptosis after combination treatment. Thus, resistant BCPAP cells were susceptible to growth-inhibitory effects of the combination treatment, although overt apoptosis was not detected at this time point.

It should be noted that the apparent discordance between the viability and apoptosis assays may also stem from the different end-points being measuring by each. The viability (trypan blue exclusion) assay detects cells with impaired integrity of plasma membrane that leads to inability to exclude cationic dyes. The apoptotic (TUNEL) assay detects cells with fragmented DNA; in the case of early- or mid- stage of apoptosis such cells have well preserved plasma membrane and do exclude trypan blue [28]. It is only at the late stage of apoptosis that integrity of plasma membrane is not preserved. A large fraction of DNA in the latter cells is fragmented to such small sections that they are extracted and not retained by fixation. Having only fractional DNA content, such cells present on the DNA content histograms as a distinct “sub-G1” subpopulation that may be TUNEL-negative [28].

Interestingly, both the resistant BCPAP cells and the inherently resistant anaplastic 8505c cells demonstrated a profound inhibition of phospho-MAPK expression. This suggests a role for metformin in interfering with signal transduction when combined with BRAFV600E inhibition and may account for decreased growth in viability assays. Resistant BCPAP cells were not susceptible to metformin alone, indicating that a rewiring process of acquired resistance may have enabled a distinct survival pathway inhibited only with combination therapy. In terms of signaling molecules, metformin also appeared to inhibit expression of total mTOR within BCPAP papillary thyroid cancer cells and increased expression of total mTOR in 8505c cells, regardless of the presence of vemurafenib. Anti-proliferative effects of metformin in BCPAP cells may be driven through diminishment of mTOR expression.

The concentration of 2 mM metformin used in this study is relatively high compared to expected human plasma levels of around 10-40 μM after receiving a standard therapeutic dose. Similar concentrations (1-20 mM) were required for AMPK activation in a prior study investigating metformin and vemurafenib in melanoma cells. It was proposed that low expression levels of OCT1 transporter in cultured cells may account for this difference, which may also be the case for our thyroid cancer cell lines [46]. Data highlights a connection of metformin to cell signaling pathways. Further research defining the wide-ranging effects of metformin at different concentrations and within different cell types is needed.

The prior investigation with melanoma found synergistic anti-proliferative effects of vemurafenib and metformin in the majority of *BRAFV600E*-positive cell lines, although some antagonistic effects were also seen [46]. In another study, a similar biguanide drug, phenformin, demonstrated cooperative effects to inhibit proliferation when combined with BRAFV600E inhibition in melanoma cells and also delayed development of resistance [47]. Although limited to an *in vitro* system, our study provides evidence that combination of targeted BRAFV600E inhibition with broader inhibition provided by metformin causes greater than additive inhibition of thyroid cancer cell growth and induction of apoptosis. Further *in vivo* studies may lead to benefits for patients with advanced *BRAFV600E*-positive thyroid cancer. This is an especially appealing combination because of the widespread use and low side effects of metformin seen in patients treated for diabetes mellitus type 2.

The second inhibitor, rapamycin, directly inhibits mTOR complex 1. Combination treatment with rapamycin and vemurafenib proved to be effective only in BCPAP papillary thyroid cancer cells, possibly due to rapamycin's more targeted mechanism of action. Resistant BCPAP cells and anaplastic 8505c cells are likely better adapted to thrive in the face of targeted inhibition due to the availability of alternative signaling pathways. It is noteworthy that the combination of rapamycin and vemurafenib resulted in very marked induction of apoptosis in BCPAP, accounting for the decrease in viability. Apoptotic cells made up 70% of all detected BCPAP cells and there was a large population of cells intensely stained with TUNEL, indicative of intensive DNA fragmentation and advanced progression of apoptosis [28]. The profound interaction of vemurafenib and rapamycin seen in BCPAP cells alone reflects cell-specific sensitivity based on the wiring pattern of BRAFV600E-mediated signal transduction. Exquisite sensitivity to combination treatment targeting BRAFV600E and a downstream integrative molecule such as mTOR may be seen in a defined population of cancer cells, as in the case of BCPAP cells.

Sensitivity of differentiated papillary thyroid cancer cells to rapamycin and vemurafenib treatment could be exploited clinically by treating with combination therapy early on in a patient's clinical course, before resistance to vemurafenib develops. Combinations of vemurafenib and mTOR inhibitors should be further studied on human cancer xenografts on immunodeficient mice, to evaluate *in vivo* effects and potential clinical benefits for treatment of advanced thyroid cancer patients.

## MATERIALS AND METHODS

### Cells and cell culture

Thyroid cell lines used in this study include a normal transformed line (Nthy-ori 3-1), a papillary thyroid cancer cell line (BCPAP), and an anaplastic thyroid cancer cell line (8505c). Dr. Norman L. Eberhardt (Mayo Clinic, Rochester, MN) generously gifted the Nthy-ori 3-1 cell line. BCPAP and 8505c cell lines were purchased from DSMZ in Braunschweig, Germany. All cell lines were cultured in Rosswell Park Memorial Institute (RPMI)-1640 supplemented with 10% fetal bovine serum (FBS), penicillin 10, 000 IU/mL, streptomycin 10, 000 μg/mL, and 2 mM L-glutamine.

### Resistant BCPAP cell line

In order to create vemurafenib-resistant BCPAP cells, cells were treated for 24 hours with increasing concentrations of vemurafenib (Chemietek, Indianapolis, IN) up to 16 μM, subcultured and allowed to grow to 70% confluence before the next treatment. Cells were treated with vemurafenib concentrations of 10 μM, 12 μM, 14 μM, and 16 μM over a period of 45 days. The resistant BCPAP cells were maintained in culture with intermittent treatments of 16 μM vemurafenib.

### Trypan blue exclusion assay

Cells were plated in six-well culture plates and allowed to adhere overnight. Each well contained 200,000 cells (8505c) or 300,000 cells (Nthy-ori 3-1, BCPAP, resistant BCPAP) in 2 mL media at the start of the experiment. Complete media was replaced with phenol-red-free RPMI containing 5% charcoal dextran-treated FBS +/− 2 mM metformin and plates were incubated for 24 hours. Cells were then treated with +/− 10 μM vemurafenib for 36 hours before viable cells were counted as cells unable to take up 0.4% trypan blue solution (Sigma). For the rapamycin experiment, the same protocol was followed except cells were treated for 24 hours with phenol-red-free RPMI containing 5% charcoal dextran-treated FBS for 24 hours and then this media was replaced with fresh media containing +/− 10 μM vemurafenib, +/− 1 μM rapamycin for 36 hours. Trypan blue exclusion assays were repeated at least once in triplicates.

### APO-BrdU tunel assay

Thyroid cells were plated in 6-well plates at 300,000 cells per well (Nthy-ori 3-1, BCPAP, and resistant BCPAP) or 200,000 cells per well (8505c) and allowed to adhere overnight. Complete media was replaced with 5% charcoal dextran-treated FBS +/− 2 mM metformin and plates were incubated for 24 hours. Cells were then treated with +/− 10 μM vemurafenib for 36 hours. Cells were collected and fixed with 1% paraformaldehyde in PBS for 1 hour on ice. Cells were washed with PBS twice and resuspended in 70% ethanol. Cells in solution were subjected to the APO-BRDU protocol (Phoenix Flow Systems, Inc., San Diego, CA). BrdU incorporated into DNA at the site of breaks was detected by anti-BrdU antibody labeled with Alexa Fluor 488 dye. Total cellular DNA was stained with DAPI. While most apoptotic cells were TUNEL-(Alexa Fluor 488)- positive, following some treatments they were also identified as the cells with fractional DNA content (“sub-G1” cells) [28, 48]. For the rapamycin experiment, the same protocol was followed except cells were treated for 24 hours with phenol-red-free RPMI containing 5% charcoal dextran-treated FBS and then media was replaced with fresh media containing +/− 10 μM vemurafenib and +/− 1 μM rapamycin for 36 hours. Intensity of cellular fluorescence was measured using a MoFlo XDP (Beckman-Coulter, Brea, CA) high speed flow cytometer/sorter. DAPI fluorescence was excited with the UV laser (355 nm), AlexaFluor 488, with the argon ion (488 nm) laser, as described earlier [49].

### Western blot

Thyroid cells were allowed to grow in T75 flasks until 70% confluence. Complete media was replaced with 5% charcoal dextran-treated FBS-containing media +/− 2 mM metformin and flasks were incubated for 24 hours before +/− 10 μM vemurafenib treatment for an additional 24 hours. After treatment, cells were collected by gentle scraping with cell scrapers, subjected to radioimmunoprecipitation assay buffer (50mM Tris-HCl[pH 7.4], 150mM NaCl, 0.2% sodium deoxycholate, 0.1% sodium dodecyl sulfate (SDS), 0.5% NP40, and 1 μM Pefabloc) and vortexed every 5 minutes for 30 minutes over ice. Lysates were centrifuged at 14,000 rpm for 30 minutes at 4°C and each supernatant was collected. Volumes containing 10 μg protein per sample were subjected to 12% SDS-polyacrylamide gel electrophoresis as in our prior studies [50, 51]. Proteins on gels were transferred to Immobilon-P membranes for 2 hours at 220 mA in a transfer chamber. Membranes were blocked with 5% milk in TBST (10 mM Tris-HCl, pH 7.5, 200 mM NaCl, 0.05% Tween-20) for 1.5 hours at room temperature and then incubated overnight in primary antibodies (Cell Signaling) at 4° Celsius. Membranes were washed three times with TBST for 5 minutes per wash and incubated with secondary antibody for 2 hours at room temperature. After 4 TBST washes of 10 minutes each, membranes were developed by enhanced chemiluminescence (Thermo Scientific) and detected on x-ray film.

### Immunofluorescence

Cell suspensions from the APO-BrdU TUNEL assay were prepared for viewing on glass slides by cytospin. Slides were viewed and images were captured using Axiovision Rel 4.8 on the Axiovert 200 M microscope (Carl Zeiss MicroImaging, Inc., Thornwood, NY).
